# A survival analysis of the timing of onset of childbearing among young females in Nigeria: are predictors the same across regions?

**DOI:** 10.1186/s12978-018-0623-3

**Published:** 2018-10-16

**Authors:** Michael O N Kunnuji, Idongesit Eshiet, Chinyere C P Nnorom

**Affiliations:** 1 Department of Sociology, University of Lagos, Lagos, Nigeria; 2 Department of Sociology/Psychology/Criminology & Security Studies, Faculty of Management & Social Sciences, Alex Ekwueme Federal University Ndufu-Alike Ikwo (AE-FUNAI), Abakaliki, Ebonyi State Nigeria

**Keywords:** Adolescent childbearing, Regional variation in Nigeria, Early marriage and cohabitation, Reproductive health, Survival analysis, Wealth

## Abstract

**Background:**

Early childbearing comes at high health costs to girls, the children they bear, their future life chances and the larger society. Nationally representative data suggest variation in onset of childbearing across regions and states of the country. Yet, there is need for strong evidence on how background characteristics explain time to first birth among young females across regions in Nigeria.

**Methods:**

We analysed the 2013 DHS dataset using Kaplan Meier and Cox Regression. The outcome variable is age at onset of childbearing with location (rural/urban), education, religion, wealth index, region and having ever married/cohabited as covariates. Models were computed for national level analysis and the six regions of the country.

**Results:**

The effect of marriage/cohabitation on time to first birth is strong and universal across the regions. Ever married girls had higher adjusted hazard ratios for starting childbearing than single girls, ranging from 5.35 in the South South to 44.62 in the North West (*p* < 0.001 in all models). Education also has significant effect on time to first birth across regions. The significance of state of residence, wealth, and religion varies across regions.

**Conclusion:**

We conclude that the combinations of factors that explain onset of childbearing vary across regions. Therefore, context specific factors should be considered in program designs aimed at achieving a significant reduction in early childbearing and similar problems in Nigeria.

## Plain English summary

When compared to adolescents within the age group of 10 and 19 years in other countries of the world, a large proportion of adolescents in Nigeria give birth to babies. The problem with adolescents giving birth is that the experience of childbearing often results in ill health for the adolescents and their babies. In addition, it affects the future of these adolescents and their babies. The adolescent mothers are often not able to continue with their education, or to acquire any meaningful training that will allow them get good jobs later in life. Previous research on the health of people in Nigeria tells us that in Nigeria, girls in certain regions, especially the northern zones are more likely to start having children than those from the southern zones, but we are yet to know whether the factors that predispose adolescents to childbearing are the same across regions. We used survival analysis to show the factors that determine how much time it takes adolescents in different regions to begin childbearing. At the national level, empirical evidence suggest that the predictors of the timing of onset of childbearing include being ever married/having ever lived with a man as if married, the zone in which an adolescent is located, how rich or poor an adolescent is, literacy and education. In the different regions, we still found that being ever married/having ever lived with a man as if married is a constant predictor of onset of childbearing. Other factors such as state of residence, how rich or poor one is, religion and education can also help us understand the onset of childbearing in some of the regions. We conclude that the combination of factors that explain onset of childbearing vary across regions and context specific factors should be considered in program designs aimed at achieving a significant reduction in early childbearing among young people.

## Background

Nigeria, the most populous country in Africa with a 2017 population of 191 million has an adolescent birth rate of 122 births per 1000 women ages 15 to 19 years [1]. About 74% of adolescent girls who give birth in the country do so at home [2] and they are exposed to many child delivery-related complications [3, 4] and poor birth outcomes [5]. Early childbearing is also a risk factor for cervical cancer [6]. In addition, research shows that early childbearing, typically before the age of 16 years, is associated with an increased risk of maternal anemia, infections, eclampsia and preeclampsia, emergency cesarean delivery, postpartum depression and inadequate breastfeeding initiation [7]. Studies further show that infants of teenage mothers are more likely to be premature and have a low birth weight [8], and are at an increased risk for respiratory distress syndrome and autism later in life [7].

Apart from these reproductive health implications, early childbearing comes at high socio-economic cost to girls and the society at large. It is associated with low educational attainment [8–10], high likelihood of staying unmarried [9, 11], marital instability, overall poor socio-economic status and welfare dependency in adult life [9–11] and lower levels of children’s education [8]. Also, it leads to stigma with psychological effects such as shame and guilt [12]. In terms of longevity, teenage mothers have lower life expectancy when compared with non-teen mothers [8]. By implication, early childbearing has negative implications for girls, their children and the society at large.

A number of studies have explored probable determinants of early childbearing, often implicating social and environmental factors. For instance, some studies show that girls living in underserved or high-poverty communities are at a high risk of beginning childbearing early [11–13] or teenage pregnancy (whether they carry the pregnancy to term or not) [14]. Early childbearing is also associated with low educational aspiration, dropping out of school and having friends, majority of whom have experienced pregnancy or are teenage mothers [11, 15] or having older sexually active siblings/pregnant or parenting sisters or having suffered sexual abuse [16]. The diversity of Nigeria which we present in the paragraph that follows explains why it is important to explore the predictors of time to first birth differently for different regions of the country.

The Nigerian population is made up of about 374 ethnic groups [2] which are politically organised into 36 states. The 36 states are often clustered into six regions/regions – North Central, North East and North West, South East, South South and South West [2] each composed of contiguous states with approximately similar ethnic groups. The North West region is home to the Hausa and Fulani Ethnic groups. In the North East region, the Kanuri, Hausa and Fulani are the majority ethnic groups but the zone is also inhabited by several smaller ethnic groups. The North Central zone is inhabited by several of Nigeria’s minority groups such as the Nupe, Tiv, Idoma and Gbagi. The majority ethnic group in the South West is the Yoruba while the Igbo are the majority ethnic group in the South East. The South South is occupied by minority ethnic groups such as the Edo, Urhobo, Itsekiri, Izon, Ibibio/Efik etc. [17]. The people in the North West and North East zones are mainly Muslims while those in the South South and South East are mainly Christians. The South West and North Central zones have mixed populations of Christians and Muslims. Generally, development indicators are better in the southern regions than the northern regions. For example, mean years of schooling are higher in the southern regions than the northern regions. On the other hand, gender inequality index is highest in the North West, followed by the North East and the North Central. Similarly, poverty is higher in the three northern regions than in any of the southern regions [18].

A recent study explained regional variation in adolescent childbearing with a model for each of the regions in Nigeria [19] focusing on first births in adolescence, as well as long after adolescence among adults in Nigeria. Consequently, the analysis includes young people and adults up to the age of 49 years. In addition, the study fails to include entry into marital union/cohabitation which is a potential predictor of onset of childbearing [20–22] and (dis)continuation of education [23, 24]. By implication, understanding adolescent childbearing requires controlling the confounding effect of entry into marriage/cohabitation. The current study of adolescents and young people includes entry into marriage or cohabitation as a covariate in explaining the onset of childbearing, contrary to the technique adopted by the mentioned previous study.

Prevalence of adolescent childbearing varies significantly across Nigeria’s regions, suggesting that region is a predictor of age at first birth [2]. Apart from region, our preliminary unadjusted national level analysis shows that factors such as living in a rural community, having little or no education, being a Muslim, and not having access to wealth also contribute to the explanation of early childbearing. Although region is a strong predictor of variance, young girls within the same region also have varied chances of becoming mothers. We ask, therefore: In the different regions of the country, what are the significant background predictors of time to first birth? Our study set out to answer this question. The danger in relying on a single explanatory model in the explanation of onset of childbearing in Nigeria is that predictive factors may differ across regions and sub-national differences may be subsumed in a national level analysis. Consequently, interventions designed on this limited understanding of the sexuality of young people across regions may fail simply because they are not context specific.

### Explaining variation in time to first birth across regions in Nigeria

A theory for regional variance in the persistence of traditional norms and resistance to international norms is found in the explanation that socio-cultural context and degree of conservatism due to religious beliefs are an important factor [25, 26]. Certain religious teachings hold the view that menarche signifies that a girl is ripe for motherhood. For instance, child-marriage is common in the Hausa-Fulani dominated Northwestern region of Nigeria where Islam is used to justify the prescription that the girl child should be married off before or at menarche to prevent premarital sex and out-of-wedlock births [27, 28]. Behind the veil of religion, however, the tradition of polygyny and poverty are major factors associated with child marriage [27, 29]. Girls married off or made to live with men under such circumstances are exposed to sex, pregnancy and childbearing. One can therefore hypothesize that entry into marriage/cohabitation will be a universal predictor of onset of childbearing.

Location may also explain time to first birth as spelt out in the metropocentric explanation [30]. The explanation holds that communities within major cities in Nigeria are better served by government in terms of provision of infrastructure and services than communities in the periphery of the state. This argument applies to access to education and reproductive health information and services which are potential predictors of reproductive health behaviour. In contexts where the theory holds, living in a rural community may reduce time to first birth as access to campaigns on the negative implications of early childbearing and access to reproductive health information and services may be limited. Against the backdrop of these explanations, we seek to identify specific predictors of time to first birth in each of the regions of Nigeria. These explanations draw from the ecological and contextual frameworks which recognise macro level factors that set the contexts that influence community, family, school and peer factors which in turn influence entry into marital unions and onset of childbearing among young females [31–33]. Ecological and contextual frameworks emphasise conceptual issues relating to the context in which reproductive health behaviour takes place and enhance the effectiveness of research and innovations on the subject matter. In the context of this study, the framework helps us explore the influence of contextual factors such as location, education, religion and entry into marital unions on the onset of childbearing.

## Methods

This study employs the 2013 Nigeria Demographic and Health Survey which is a nationally representative study that administered a structured questionnaire to 17,359 men and 38,948 women (15–49 years) across the 36 states and Federal Capital Territory of Nigeria. The survey covers a wide range of topics, including fertility, entry into marital union, reproductive health, domestic violence etc. We extracted the data for all the young females (15–24 years), a total of 14,619 respondents included in the study for the purpose of this analysis. Details about the DHS sampling techniques and sample size are available at http://www.dhsprogram.com/. The Nigeria DHS research protocol complies with the WHO ethical and safety recommendations and the National Health Research Ethics Committee guidelines. We did not gather primary data for the study, so we did not require any further ethical approval outside the approval obtained by the National Population Commission and ICF International.

This is a time-to-event analysis which models time (in years) until the event of first childbirth [34, 35]. We used the Kaplan-Meier test to estimate mean ages at first birth for different categories of young females. Kaplan-Meier survival estimate is mathematically denoted by

$$ S\left({t}_j\right)=S\left({t}_{j-1}\right)\left(1-\frac{d_j}{n_j}\right). $$

The computation represents the cumulative probability of not having started childbearing at a given time *t*_*j*_, where *n*_*j*_ is the number of young females yet to begin childbearing just before *t*_*j*_ and *d*_*j*_ the number of first childbirths at *t*_*j*_. We present estimated mean ages for categories of young females and use the Log Rank test to compare means for significant difference. Survival functions for background characteristics are presented in charts.

We used the Cox proportional hazards models to estimate the probability that an individual will have first childbirth around a particular point in time. The model is written as

$$ h(t)={h}_0(t)\times \exp \left\{{b}_1{x}_1+{b}_2{x}_2+\dots +{b}_p{x}_p\right\}. $$

Adjusted hazard ratios comparing young females of different categories with reference groups are presented and flagged where they are statistically different from the reference categories in seven models, the first for the entire national sample of young females and one for each of the six regions. Age at first child birth was used as the Time variable while having had a child is the event of interest. Covariates included in the analysis are place of residence (rural/urban), highest educational qualification, religion, being ever married/having ever cohabited, wealth index and state. In the national level analysis, region was used instead of state. All the covariates are categorical and the first category was used as the reference category in all the models.

## Results

The background characteristics of the study participants are presented in Table 1. As the table shows, about 60% of the respondents were drawn from the rural parts of Nigeria, 54% of them had secondary education (about 12 years of formal education) and 56% were single/never married. The table further shows that 36% of them had begun having children.

**Table 1:** Background characteristics of respondents by region

	North Central	North East	North West	South East	South South	South West	Nigeria
	*N* = 2432 (%)	*N* = 2531 (%)	*N* = 3580 (%)	*N* = 1647 (%)	*N* = 2439 (%)	*N* = 1990 (%)	*N* = 14,619 (%)
Location							
Urban	823 (33.8)	660 (26.1)	935 (26.1)	1115 (67.7)	844 (34.6)	1454 (73.1)	5831 (39.9)
Rural	1609 (66.2)	1871 (73.9)	2645 (73.9)	532 (32.3)	1595 (65.4)	536 (26.9)	8788 (60.1)
Education							
No education	410 (16.9)	1260 (49.8)	2225 (62.2)	11 (0.7)	28 (1.1)	91 (4.6)	4025 (27.5)
Primary	382 (15.7)	377 (14.9)	444 (12.4)	166 (10.1)	335 (13.7)	154 (7.7)	1858 (12.7)
Secondary	1464 (60.2)	810 (32.0)	849 (23.7)	1347 (81.8)	1928 (79.0)	1511 (75.9)	7909 (54.1)
Higher	176 (7.2)	84 (3.3)	62 (1.7)	123 (7.5)	148 (6.1)	234 (11.8)	827 (5.7)
Religion							
Catholics	312 (13.1)	83 (3.3)	80 (2.3)	801 (48.8)	239 (9.9)	87 (4.4)	1602 (11.1)
Other Christians	1004 (42.1)	454 (18.1)	160 (4.5)	836 (50.9)	2138 (88.3)	1289 (65.2)	5881 (40.7)
Muslims	1070 (44.8)	1978 (78.6)	3287 (93.2)	4 (0.2)	44 (1.8)	600 (30.4)	6983 (48.3)
Wealth quintile							
Poorest	176 (7.2)	782 (30.9)	1122 (31.3)	72 (4.4)	13 (0.5)	25 (1.3)	2190 (15.0)
Poorer	437 (18.0)	731 (28.9)	1117 (31.2)	236 (14.3)	237 (9.7)	143 (7.2)	2901 (19.8)
Middle	780 (32.1)	466 (18.4)	607 (17.0)	427 (25.9)	665 (27.3)	303 (15.2)	3248 (22.2)
Richer	550 (22.6)	332 (13.1)	456 (12.7)	507 (30.8)	854 (35.0)	659 (33.1)	3358 (23.0)
Richest	489 (20.1)	220 (8.7)	278 (7.8)	405 (24.6)	670 (27.5)	860 (43.2)	2922 (20.0)
Ever/never married							
Single never married	1504 (61.8)	920 (36.3)	995 (27.8)	1348 (81.8)	1873 (76.8)	1546 (77.7)	8186 (56.0)
Ever married/cohabited	928 (38.2)	1611 (63.7)	2585 (72.2)	299 (18.2)	566 (23.2)	444 (22.3)	6433 (44.0)
Has begun childbearing							
No	1664 (68.4)	1288 (50.9)	1715 (47.9)	1322 (80.3)	1764 (72.3)	1543 (77.5)	9296 (63.6)
Yes	768 (31.6)	1243 (49.1)	1865 (52.1)	325 (19.7)	675 (27.7)	447 (22.5)	5323 (36.4)

### Estimated mean ages at first birth

The results presented in Table 2 show that the Kaplan-Meier estimated mean age at first birth is higher in urban areas than rural areas (21.83 vs 19.67), and among single/never married young females than those who had ever married/cohabited (23.35 vs 18.33). The results further reveal that the mean age at first birth increases with education and wealth index. For the different regions, the estimated mean ages at first birth range from 18.81 years in the North West to 22.29 years in the South East. The Cox regression model shows that only education, wealth index, having ever married/cohabited, and region are significant predictors of time to first birth at the national level when background characteristics are adjusted for as shown by the adjusted hazard ratios in Table 2.

**Table 2:** Summary of Kaplan-Meier survival tests & Adjusted hazard ratios for age at first birth

	Event	Summary of Kaplan-Meier tests	Adjusted Hazard ratios
	Began childbearing (%)	Estimated mean age (in years) at first birth (95% CI)	HR (95% CI)
Location			
Urban (RC)	1395 (23.9)	21.83 (21.73–21.92)	
Rural	3928 (44.7)	19.67 (19.58–19.75)	1.06 (0.98–1.14)
Log Rank (Mantel-Cox) Chi-square		991.03 (*p* < 0.001)	
Education			
No education (RC)	2539 (63.1)	18.03 (17.93–18.14)	
Primary	907 (48.8)	19.16 (18.98–19.34)	0.96 (0.88–1.05)
Secondary	1772 (22.4)	21.71 (21.63–21.79)	0.70 (0.64–0.77)^***^
Higher	105 (12.7)	23.38 (23.26–23.50)	0.36 (0.29–0.45)^***^
Log Rank (Mantel-Cox) Chi-square		3703.39 (*p* < 0.001)	
Religion			
Catholics (RC)	367 (22.9)	21.94 (21.77–22.12)	
Other Christians	1503 (25.6)	21.63 (21.53–21.72)	1.06 (0.94–1.19)
Muslims	3383 (48.4)	19.30 (19.20–19.39)	0.98 (0.86–1.13)
Log Rank (Mantel-Cox) Chi-square		1371.861 (*p* < 0.001)	
Wealth quintile			
Poorest (RC)	1260 (57.5)	18.16 (18.00–18.32)	
Poorer	1429 (49.3)	19.17 (19.02–19.32)	0.89 (0.83–0.96)^**^
Middle	1169 (36.0)	20.50 (20.36–20.64)	0.86 (0.79–0.94)^**^
Richer	964 (28.7)	21.38 (21.26–21.51)	0.72 (0.65–0.81)^***^
Richest	501 (17.1)	22.53 (22.42–22.64)	0.56 (0.49–0.64)^***^
Log Rank (Mantel-Cox) Chi-square		2199 (*p* < 0.001)	
Ever/never married			
Single never married	450 (5.5)	23.35 (23.29–23.41)	
Ever married/cohabited	4873 (75.8)	18.33 (18.25–18.40)	10.60 (9.56–11.75)^***^
Log Rank (Mantel-Cox) Chi-square		5813.19 (*p* < 0.001)	
Region			
North Central	768 (31.6)	21.11 (20.96–21.27)	
North East	1243 (49.1)	19.26 (19.10–19.42)	1.24 (1.12–1.37)^***^
North West	1865 (52.1)	18.81 (18.68–18.95)	1.21 (1.10–1.33)^***^
South East	325 (19.7)	22.29 (22.14–22.45)	1.02 (0.88–1.17)
South South	675 (27.7)	21.36 (21.21–21.52)	1.45 (1.29–1.62)^***^
South West	447 (22.5)	21.96 (21.80–22.11)	1.14 (1.01–1.29)^*^
Log Rank (Mantel-Cox) Chi-square		1777.26 (*p* < 0.001)	

* *p* < 0.05, ** *p* < 0.01, *** *p* < 0.001

*HR* Hazard ratio, *RC* Reference Category, *CI* Confidence Interval

**Fig. 1:**
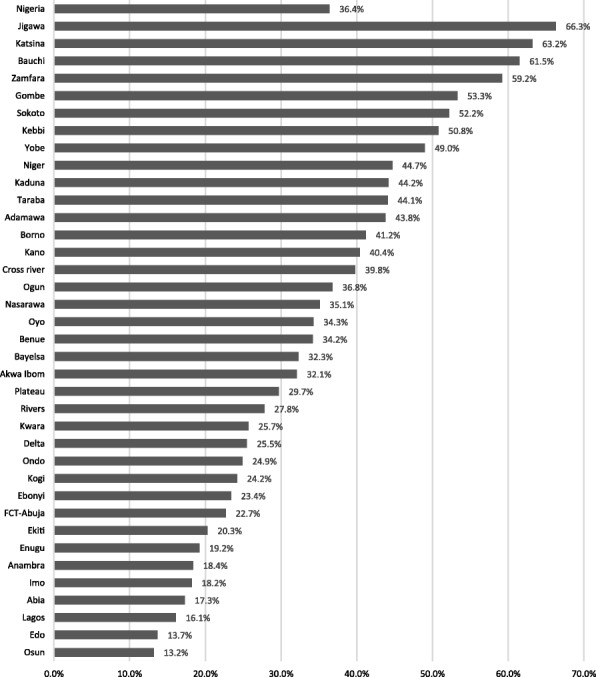
Proportions of young females (15–24 years) who have begun childbearing by state

The North East, North West and South South have significantly higher adjusted hazard ratios (1.24, 1.21 and 1.45, *p* < 0.001) than the North Central region as shown in Table 2. The South West also has a marginally higher adjusted hazard ratio (1.14, *p* < 0.05). Figure 1 further shows the variableness of the proportion of young females who have begun childbearing across the states of the federation. The proportion is least in Osun state (13.2%) and highest in Jigawa state (66.3%). In the whole of Nigeria, 36.4% have begun childbearing. The highest proportions of young females who have begun childbearing are found in the North East and North West states of Jigawa, Katsina, Bauchi, Zamfara and Gombe.

**Fig. 2:**
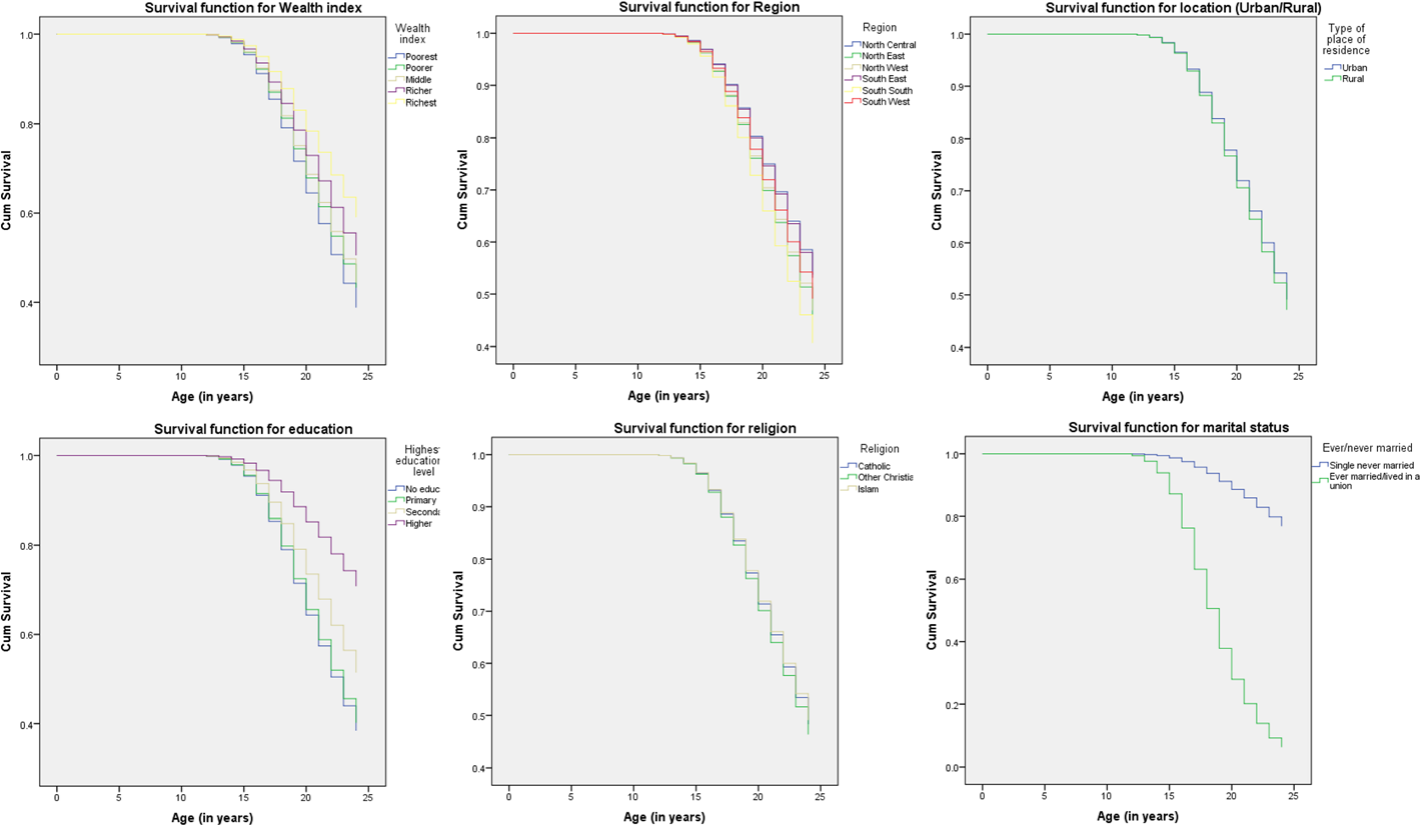
Survival functions for background characteristics

Figure 2 shows the survival functions for wealth index, region, location, education, religion and marital status. The charts suggest that having ever married/cohabited, education and wealth have the most effect on time to first birth.

### Regional variation in predictors of time to first birth

Predictors of time to first birth vary across regions. In the North Central region, young females with secondary education have a lower hazard ratio for beginning childbearing (0.75, *p* < 0.01) compared to those with no formal education in Table 3. Girls in the Richer and Richest quintiles also have lower hazard ratios for beginning childbearing (0.66, *p* < 0.05; 0.51, *p* < 0.01). Ever married/cohabited girls have a hazard ratio 20.37 times that of single/never married girls. The analysis also shows that states are significantly different within the region. In the North East, having a secondary or higher education reduces the likelihood of beginning to have children at any given time within the first 24 years of life. The effect of wealth is, however, marginal and inconsistent. The hazard ratio for ever married/cohabited girls is 11.31 times higher than the hazard ratio for single girls (*p* < 0.001). States within the region also have varying effects on time to first childbirth. In the North West, education, wealth and religion have significant effect on time to first child birth. Lower hazard ratios were found for girls with secondary (0.63, *p* < 0.001) and higher education (0.41, *p* < 0.05). Girls from higher wealth quintiles also had lower adjusted hazard ratios when compared with girls in the Poorest quintile. Ever married/cohabited girls have an adjusted hazard ratio that is 44.62 times that of never married girls.

**Table 3:** Adjusted hazard ratios for age at first birth by region

	North Central	North East	North West	South East	South South	South West
	HR (95% CI)	HR (95% CI)	HR (95% CI)	HR (95% CI)	HR (95% CI)	HR (95% CI)
Location						
Urban (RC)						
Rural	0.97 (0.78–1.20)	1.00(0.84–1.21)	0.97 (0.82–1.15)	1.25 (0.96–1.63)	1.01 (0.83–1.23)	1.10 (0.84–1.44)
Education						
No education (RC)						
Primary	0.99 (0.79–1.24)	0.89 (0.75–1.06)	0.96 (0.83–1.12)	1.22 (0.43–3.49)	1.17 (0.69–1.97)	1.16 (0.78–1.72)
Secondary	0.75 (0.60–0.93)^**^	0.67 (0.55–0.81)^***^	0.63 (0.52–0.77)^***^	0.91 (0.333–2.53)	0.85 (0.51–1.42)	0.84 (0.57–1.24)
Higher	0.68 (0.42–1.11)	0.27 (0.14–0.51)^***^	0.41 (0.20–0.86)^*^	0.26 (0.72–0.92)^*^	0.30 (0.14–0.62)^**^	0.46 (0.28–0.77)^**^
Religion						
Catholics (RC)						
Other Christians	1.14 (0.88–1.48)	0.94 (0.63–1.40)	0.34 (0.21–0.55)^***^	0.99 (0.78–1.25)	1.46 (1.08–1.96)^*^	1.25 (0.73–2.12)
Muslims	1.06 (0.78–1.43)	0.89 (0.60–1.32)	0.38 (0.26–0.57)^***^	0.87 (0.32–2.42)	1.36 (0.70–2.63)	1.11 (0.63–1.94)
Wealth quintile						
Poorest (RC)						
Poorer	0.99 (0.74–1.32)	0.96 (0.83–1.10)	0.86 (0.78–0.96)^**^	0.54 (0.33–0.90)^*^	0.76 (0.24–2.43)	0.47 (0.25–0.89)^*^
Middle	0.91 (0.68–1.22)	0.82 (0.68–0.99)^*^	0.77 (0.666–0.89)^**^	0.75 (0.46–1.22)	0.75 (0.24–2.37)	0.64 (0.34–1.23)
Richer	0.66 (0.47–0.92)^*^	0.81 (0.64–1.03)	0.82 (0.65–1.04)	0.53 (0.33–0.87)^*^	0.55 (0.18–1.75)	0.53 (0.27–1.03)
Richest	0.51 (0.34–0.77)^**^	0.69 (0.48–0.99)^*^	0.69 (0.50–0.97)^*^	0.47 (0.27–0.80)^**^	0.37 (0.12–1.20)	0.44 (0.21–0.90)
Ever/never married						
Single never married						
Ever married/cohabited	20.37 (14.69–28.25)^***^	11.31 (8.35–15.31)^***^	44.62 (25.06–79.46)^***^	10.55 (8.08–13.757)^***^	5.35 (4.53–6.32)^***^	12.66 (9.57–16.75)^***^
State^a^						
Reference state						
State 2	0.84 (0.61–1.15)	1.02 (0.82–1.28)	1.13 (0.95–1.35)	0.90 (0.63–1.29)	1.92 (1.37–2.68)^***^	0.80 (0.56–1.15)
State 3	0.73 (0.56–0.95)^*^	1.27 (1.02–1.58)^*^	1.18 (0.99–1.40)	1.32 (0.91–1.90)	2.05 (1.50–2.81)^***^	1.00 (0.69–1.45)
State 4	0.66 (0.49–0.89)^**^	1.16 (0.95–1.42)	1.11 (0.93–1.31)	0.89 (0.57–1.39)	1.46 (1.04–2.03)^*^	1.15 (0.82–1.61)
State 5	0.85 (0.6–1.17)	1.41 (1.17–1.69)^***^	0.94 (0.79–1.11)	0.94 (0.63–1.40)	2.26 (1.68–3.05)^***^	0.94 (0.65–1.36)
State 6	0.83 (0.62–1.10)	1.37 (1.10–1.71)^**^	1.26 (1.03–1.53)^*^		1.48 (1.08–2.02)^*^	1.17 (0.83–1.65)
State 7	0.75 (0.57–0.98)^*^		0.93 (0.77–1.11)			

*HR* Hazard Ratio, *RC* Reference Category, *CI* Confidence Interval

* *p* < 0.05, ** *p* < 0.01, *** *p* < 0.001

^a^ North Central states: Ref state – Niger, State 2 – FCT, State 3 – Nasarawa, State 4 – Plateau, State 5 – Benue, State 6 – Kogi, State 7 – Kwara

North East states: Ref state – Yobe, State 2 – Borno, State 3 – Adamawa, State 4 – Gombe, State 5 – Bauchi, State 6 – Taraba

North West states: Ref state – Sokoto, State 2 – Zamfara, State 3 – Katsina, State 4 – Jigawa, State 5 – Kano, State 6 – Kaduna, State 7 – Kebbi

South East states: Ref state – Anambra, State 2 – Enugu, State 3 – Ebonyi, State 4 – Abia, State 5 – Imo

South South states: Ref state – Edo, State 2 – Cross River, State 3 – Akwa Ibom, State 4 – Rivers, State 5 – Bayelsa, State 6 – Delta

South West states: Ref state – Oyo, State 2 – Osun, State 3 – Ekiti, State 4 – Ondo, State 5 – Lagos, State 6 – Ogun

In the South East region, lower hazard ratios are associated with higher wealth. Having higher education also significantly affects time to first birth, with an adjusted hazard ratio of 0.26 (*p* < 0.05). The adjusted hazard ratio for ever married/cohabited young females is 10.55 times that of single/never married girls. No state has an adjusted hazard ratio different from the reference state. In the South South on the other hand, all the states have significantly higher adjusted hazard ratios for beginning childbearing when compared to the reference state (Edo) and girls with higher education have a significantly lower adjusted hazard ratio (0.30, *p* < 0.01) than girls with no formal education. Having ever married/cohabited has the strongest effect on time to first birth, with an adjusted hazard ratio of 5.35 (*p* < 0.001). In the South West, having a higher education significantly increases time to first birth in comparison with girls with no formal education (*p* < 0.01). Ever married/cohabited girls have an adjusted hazard ratio of 12.66 (*p* < 0.001).

## Discussion

Having ever married/cohabited is a universal and strong predictor of time to first birth across all six regions of the country. This confirms findings from earlier studies that living in marital unions or cohabiting exposes girls to pregnancy and childbearing [20–22, 36–38]. A major implication of this finding is that all interventions that seek to reduce early childbearing among adolescents need to also seek to stop the practice of child marriage and cohabitation. It also stresses the need for the adoption and implementation of the Child Rights Act/Law across states in Nigeria. The law prohibits any form of marriage or cohabitation with persons under the age of 18 years. Implementing this law will protect children who are below the age of consent from adults who wish to ‘marry’ them or cohabit with them.

The second most import factor in time to first birth is education. In all three northern regions, having secondary education has a significant effect on time to first birth while in the southern regions, only higher education significantly affects time to first birth. This supports earlier studies that associate early onset of childbearing with poor education [11, 15, 39] even though it does not address the problem of temporal sequence as we are not able to determine whether low educational aspiration drives adolescent childbearing or vice versa. The current study shows that even when marriage or cohabitation is held constant, education continues to have a significant effect on time to first birth. There is need, therefore, for the full implementation of the universal basic education policy that requires that the first 9 years of education be free and compulsory for all in Nigeria. The implementation of this policy has the potential of increasing reproductive health knowledge and outcomes.

Wealth also has significant influence on time to first birth in all but one of the six regions, albeit, with some inconsistency in some regions. Generally speaking, however, the likelihood of starting to have children is lower for all the four top quintiles than for the Poorest quintile in all the regions even though the result is not significant in some cases. This finding is in agreement with earlier studies [39]. One possible explanation for the role of wealth in time to first birth is that poverty reduces access to sexual and reproductive health knowledge and services [40] which may in turn predict adolescent childbearing. In addition, females from poor households may be involved in relationships with older, richer men for survival [41]. The implication of this finding for programming is that an ecological approach to the problem of early childbearing is required. The provision of sexual and reproductive health information and services should be reinforced with better economic conditions that reduce the lure of relationships with older, richer men.

Generally, young females in the northern states begin to have children earlier. This supports the position in the literature [25, 26]. It is, however, interesting to note that state of residence is also a source of variation in time to first birth in contexts that are assumed to be largely homogeneous. Only the South East and South West regions have states that are homogeneous in respect to time to first birth among young females. In the other regions, we observed state level variations, contrary to the assumption that regions approximate socio-cultural clusters with a measure of internal homogeneity. We argue that variation in sub-national political administration makes a difference in time to first birth. This shows the need for a study to document the effect of state level administration on youth sexual and reproductive health in general, and exposure to pregnancy and childbearing in particular. Such context-specific study will focus on what state governments are doing differently in terms of policy adoption and implementation, state level laws, and the activities of non-government organizations and how these affect the onset of childbearing among young people. The South South represents a classic case of variation with all the states having adjusted hazard ratios that differ significantly from the reference state (Edo). This region requires a state level analysis.

### Limitations of the study

The survey is based on self reports which may be inaccurate due to poor recall of events. The choice of covariates in the analysis is also limited to background characteristics. There is the possibility, however, that other variables not included in the analysis significantly affect time to first birth. This study is also limited in scope. For instance, it is desirable to have state-level analyses but this is outside the scope of this study.

## Conclusion

The negative health implications of early onset of childbearing stress the importance of evidence-based interventions. Interventions that will yield positive results need to target adolescents at high risk of early marriage, and those living in extreme poverty. While in-school adolescents may have access to some sexuality education, those not registered in schools may be left out and may have limited access to reproductive health information and services. Interventions should therefore seek to address the specific needs of out-of-school girls. Such interventions must also be multi-level in nature, in order to address contextual factors that reinforce the belief in marrying girls off early at the community and national levels. In addition, the most hit states identified in this study should be prioritized in programming. While it may be convenient for governments and non-governmental organizations to pilot their interventions in cities, addressing the problem of adolescent childbearing and similar problems may be more successful if the worst hit states are prioritized.

The concentration of strong non-governmental organizations in cities like Lagos, Abuja, Kano, Calabar and Port Harcourt often gives those cities too much attention at the expense of many less developed states. In the North West for instance, the proportion of young females who have begun childbearing is lowest in Kano state which has a greater likelihood than any of the other states to attract intervention programs because of its relative cosmopolitan nature and population. Yet, Jigawa, Katsina, Bauchi, Zamfara and Kebbi states require greater attention, especially those households living in extreme poverty in these states. The findings of this study also have wider implications within the Nigerian context. Addressing similar reproductive health problems such as youth uptake of human immune-deficiency virus testing and unsafe sex also require an approach that prioritizes the worst hit states of the country, and the poorest of the poor in such states.

## Abbreviations

CI: Confidence interval
HR: Hazard ratio
RC: Reference category
